# Supplementary material for: Are carbon emissions trading and green financial instruments synergistic? -Comprehensive quantitative research based on content analysis
Source: PLoS One. 2024 Mar 7;19(3):e0298601. doi: 10.1371/journal.pone.0298601 (PMC10919601; doi:10.1371/journal.pone.0298601)
Supplement: S1 Data — (ZIP) [file pone.0298601.s001.zip › Data/地级市Z_F工作报告.zip!/┴╔─■/║∙┬1⁄2╡║╩╨/└┤╫╘╣1⁄2╓┌║┼í╛┬φ┐╦╩2╛▌═°í┐_2015.docx]

各位代表：

　　现在，我代表市人民政府向大会报告工作，请予审议，并请各位政协委员和其他列席同志提出意见和建议。

　　一、2014年工作回顾

　　2014年，是我市发展史上极不平凡的一年。我市经济发展进入“三期叠加”时期，面临经济下行和特大旱灾双重压力，困难加大，挑战增多。一年来，在市委的正确领导下，全市人民团结拼搏，锐意进取，改革创新，经济指标保持合理增长，社会事业持续繁荣发展，人民幸福指数稳步提升，完成了市五届人大一次会议确定的各项任务。

　　预计2014年，全市地区生产总值800亿元，增长4.5%;规模以上工业增加值180亿元，增长7%;公共财政预算收入81.6亿元，下降7.7%;固定资产投资568亿元，下降10%;社会消费品零售总额404亿元，增长12%;实际利用外资5.5亿美元，下降13.5%;引进省外内资223亿元，增长18%;出口总额12.8亿美元，增长15.8%;城镇居民人均可支配收入22926元，增长9%;农村居民人均可支配收入9609元，增长10%。

　　2014年，我市的公共财政预算收入、固定资产投资两项指标明显下滑，主要原因：一是客观上，宏观经济持续低迷，老工业基地尚未根本解决的体制和机制性矛盾集中爆发，房地产业不景气，长期积累的产业结构不合理问题集中显现;二是政策上，贯彻落实国家和省对企业减免各种税费等政策，扩大了“营改增”范围;三是主观上，坚持实事求是组织财政收入，统计真实性项目投资，做实了发展基础。尽管如此，我市主要经济指标增速在全省的位次不降反升，经济运行仍处于合理区间。同时，就业和民生增势明显，居民收入实现较快增长，保证了经济发展的质量和效益。

　　一年来，我们主要取得了九个方面的业绩：

　　1、各项改革加速推进。行政体制改革逐步提速。完成卫生计生部门整合，调整工商质监体制，重组食品药品监督管理机构。取消、下放行政权力事项263项，废止文件51个。推进财税体制改革。实施市本级零基预算改革，部门预决算和“三公”经费预决算全面公开。扩大“营改增”范围，缓征、取消8项行政事业性收费和政府性基金，减轻企业负担1.2亿元，受益企业3329家。深化农村改革。土地承包经营权确权登记15万亩，土地承包经营权流转54.7万亩。推进工商登记制度改革，新增登记企业2.2万家，增长1倍。公司认缴注册资本113.3亿元，增长350%。中冶葫芦岛有色股份制改革顺利实施，锌业股份恢复上市交易，宏跃集团与江西铜业成功合作。

　　转型发展取得突破。抢抓新一轮东北振兴机遇，在“央企辽宁行”活动中对接项目15个、总投资1000亿元，在“知名民企辽宁行”活动中推进合作项目5个、总投资523亿元。启动城区老工业区搬迁改造工作，改造方案通过国家审批，连山老工业区列入东北四省区国家首批支持范围。

　　2、工业经济和民营经济平稳运行。深入实施工业强市战略。出台35条稳增长、促投资措施，提升企业效益。规模以上工业总产值953亿元，增长5.5%。6个产业集群全部成为省重点，销售收入1132.7亿元，增长8.3%。国家级高新技术企业达到25家。锦西石化国Ⅳ标准汽油、方大化工烧碱完成技改升级。聚氨酯产业集群销售收入突破500亿元，泳装成为省服装百亿产业集群。

　　启动建设“龙湾硅谷”。软件产业园入驻企业27家。渤船重工博士后工作站成立，打渔山泵产品检测中心运营。孚迪斯高温航空润滑油、宏兴氧化脱硝技术填补国内空白，中航黎明带式喂料机获中国专利优秀奖，长志泵业化工离心泵等17项产品获省“专精特新”产品，渤海钢管等19家企业成为省级创新型中小企业。

　　扎实开展项目年活动。开工建设亿元以上项目310个，总投资2663亿元，当年完成投资410亿元。投资12亿元的渤海船舶产业园、投资10亿元的辽宁合众凯达20万吨铜铝复合排一期等55个项目竣工。辽宁康乃尔MDI项目可研报告编制完成，渤船海工填海工程完工。大唐热电项目环评通过国家核准，徐大堡核电成为国家东北振兴支持项目。

　　民营经济健康发展。民营经济增加值350亿元，增长7%。扩展“助保贷”范围，帮助中小微企业贷款8150万元，增强了民企发展动力。伊菲科技在新三板挂牌，概思能源在辽宁股权交易中心挂牌。308家个体工商户实现“个转企”，1000家中小微企业开展网上营销，订单增长20%。兴城粉末冶金等10家企业成为省“守合同重信用”企业。

　　3、对外开放深度推进。加快招商选资。引进高端医疗、房车生产等外资项目25个，投资额9亿美元;引进80万吨MDI等省外内资项目19个，投资额668亿元。对外投资与合作实现重大突破。兴城泳装在德国法兰克福和美国洛杉矶建立营销平台，长志泵业在德国慕尼黑投资建立流体技术公司，七星钢管与文莱企业、金地矿业与香港利华分别签订合作协议，康乃尔聚氨酯公司成功并购瑞典凯美拓投资公司。锦西石化恢复出口，泳装和船舶出口基地被评为省级出口基地。成功举办第四届泳博会和第四届泵博会，对外开放迈出了坚实的步伐。

　　深入推进园区建设。园区承载力增强。118平方公里起步区实现“七通一平”;绥中港2个5万吨级泊位建成通航，柳条沟港4个5万吨级泊位主体完工，兴城国家一级渔港建成使用。推动复合型园区建设。东戴河·中关村科技产业园投融资等4个规划通过评审，金辰中小企业园标准化厂房主体完工，环球之星工业园启用，东北泵阀城引进项目15个。港区一体化实现突破。绥中港物流产业园总体规划通过评审，疏港铁路通车。柳条沟港恒大二期等临港物流基地建成使用。

　　4、县域经济稳步发展。投入抗旱资金2.6亿元，举全市之力战胜了有气象记录以来最严重的特大旱灾，抗旱工作受到国务院副总理汪洋的表扬。县域地区生产总值实现397亿元，增长5%;公共财政预算收入38.2亿元，增长4.1%。蔬菜、肉蛋奶和水产品产量分别增长5%、5.3%和2.2%。

　　农业综合效益逐步显现。农业产业化加快推进。设施蔬菜、节水滴灌、粮油高产示范区和现代海洋牧场“4个20万亩”工程超额完成，新植经济林15.9万亩，九股河食品、豪远蓝莓等5个农产品深加工项目投产。现代农业取得成效。新增家庭农场36家、农民专业合作社400家，国家级合作示范社7家。新认证绿色食品30个、无公害农产品25个，国家地理标志商标达到19件，名列全国30强。兴城红崖子花生列入欧盟互认地理标志保护产品。治理水土流失33万亩，新建水源工程1354项，保障了6.9万农村居民饮水安全。兴城荣获全国平安渔业示范县。

　　5、服务业新兴业态迸发活力。实施服务业发展四年行动计划，当年安排5000万元专项资金，争取上级资金1亿元，推动服务业加快发展。2014年，服务业增加值323.1亿元，增长3.5%，占全市地区生产总值的40.2%。

　　服务业集聚区扩容发展。全市集聚区营业收入400亿元，增长8%。完成工业企业主辅分离35家，新增商贸物流企业12家。连山东城商业金廊开工建设，建昌辽西农产品交易中心、兴城大红门物流商贸城、龙港恒嘉国际家居广场、南票尚海湾商贸城主体完工。兴城商贸旅游集聚区成为省级服务业集聚区。成功引进广发银行、阜新银行，葫芦岛农商银行组建运营，全市银行业金融机构达到22家，批筹开业小贷公司29家，本外币存款余额突破1300亿元，贷款余额突破800亿元。

　　电子商务跨越发展。我市被确定为国家跨境电子商务出口试点城市，辽宁工程技术大学成立电子商务研究机构。电子商务大厦和泳业国际仓储中心投入运营。全市网商网店7500家，交易额50亿元，泳装网销量占全国的70%。全国首家泳装跨境贸易电子商务平台通关上线，打开了物流产业发展新通道。

　　文化旅游产业提质升级。旅游环境不断优化。大觉华寺复建奠基，百大温泉水上乐园开工建设，丽汤温泉、汤上温泉部落主体竣工，比基尼广场会展中心建成使用。与辽冀蒙八城市结成旅游联盟，成功举办首届菩提文化旅游博览会，龙潭大峡谷取得国家地质公园资格，觉华岛成为4A级景区和中国最佳佛教文化旅游目的地，绥中被评为中国最美休闲度假名县。文化建设取得成果。前所镇跻身中国历史文化古镇，针刺葫芦《三顾茅庐》获省文化创意金奖，泳博会成为省文化产业“一县一品牌”示范项目。全市旅游人数、旅游总收入均增长25%。

　　6、基础设施和生态文明建设不断加强。城市基础设施逐步完善。新建和改造供水管线3.9万米，改造升级老旧供暖管网80公里。兴建高速公路竣工通车，实现了我市县县通高速的目标。完成102线、306线大修工程，东戴河动车站建成通车。天化路改造启动建设，新增城市公交线路3条，更新新能源公交车77台，“六线四环”城区公交格局基本形成。青山水库具备供水能力，猴山水库成为东北振兴重点项目。开展“我们的乡村·我们的家园”宜居乡村建设活动，287个行政村成为首批宜居达标建设村。

　　生态建设持续提升。造林绿化46.9万亩，森林覆盖率36.2%，城区绿化覆盖率42.2%，绿地覆盖率41.1%，创森工作通过国家预核检，虹螺山晋升为国家级自然保护区。青山、碧水、蓝天工程深入实施，治理矿山地质环境2122亩，兴城河、黑水河等4条河流完成整治，近岸海域水质符合国家标准。兴城河口湿地公园开工建设，碣石、觉华岛成为国家海洋公园，兴城荣获全国湿地保护先进县。高新区等4家污水处理厂试运行，污水处理合格率98.7%。杨家杖子被确定为全省可持续发展试验区，建昌省级生态县创建通过评估，兴城被评为中国宜居小城，我市跻身全国“绿色肺活量”城市20强。

　　7、民生质量不断提高。坚持把保障和改善民生放在首位，坚持财政支出向民生倾斜，争取上级资金82亿元，增长22.4%，全部用于基本民生和重点民生工程，市政府承诺的10件民生实事全部完成。新增实名制就业4.4万人，完成省定指标的110%。困难家庭大学毕业生就业率100%，零就业家庭动态管理为零，城镇登记失业率3.24%。城乡低保标准分别提高10.5%和10.4%，14.9万城乡群众受益。提高事业单位中高级专业技术人员津补贴标准，超过全省平均水平。启动城镇居民大病保险，城乡大病保险全面实施。新农合常住人口参合率99.1%，基金到位率100%。关心老年人生活，14.8万企业退休人员养老金提高11.5%。改扩建6所农村中心敬老院，开工建设500张床位的老年公寓，给予80至89岁低收入老人每人每月100元高龄津贴。新建保障性住房800户，廉租房租赁补贴200户，改造农村危房2235户。免收普通高中孤儿学费，资助家庭困难高中学生9400人次。实施“两癌”筛查，免费为3.2万农村贫困妇女进行检查。实现粮食作物病虫害专业化防治，完成280万亩统防统治。推动粮食作物农业保险，自愿参保农户实现全覆盖。实施扶贫开发项目98个，7.1万贫困人口收入达到3500元，超出省定指标9.4%。农村亮化工程完工，为全市行政村安装路灯1.5万盏。实施“天眼工程”，安装治安监控摄像头6760个，群众安全感不断提升。

　　8、社会事业全面进步。引进海外研发团队5个，国家级科普示范基地达到5家，获得省级科技进步奖2项。教育事业均衡发展。新建乡镇标准化幼儿园37所，改造各类学校79所，实验高中被评为省特色普通高中，渤海船院成为国家示范性高职院校。北京语言大学国际教育园区开工建设，渤海船院新校区、辽宁省实验中学东戴河校区建成使用，中高等教育园区已经形成。卫生事业长足进步。市中心医院龙湾院区竣工使用，并荣膺鲁班奖。建成社区卫生服务中心30家，社区卫生服务机构覆盖率95%，基本药物制度乡镇覆盖率100%。落实“单独两孩”生育政策，人口结构得到优化。文化活动丰富多彩。成功举办读书节、文化大讲堂、群众文化节和沙滩音乐季系列活动，音乐剧《因为爱》获省政府文华奖4项大奖。整合成立市群众艺术馆，连山古玩文化街揭牌运营，创建省级文化乡镇(街道)、村(社区)58个。竞技体育成果显著，在省“十二运”获得21.45枚金牌，第八届省民族运动会喜获六金。

　　民族关系和谐稳定，宗教事务健康发展。龙港区被国务院评为民族工作先进集体。国防动员、军民共建和民兵预备役工作成绩显著，国家安全、国土资源、人防地震、妇女儿童、新闻出版、红十字会和慈善等工作不断加强，监察、统计、物价、质监、档案、地方志等工作取得了新成绩。

　　9、精神文明和民主法制建设深入推进。围绕“两个一百年”目标和中国梦主题，广泛开展社会主义核心价值观宣传教育，我市成为创建全国文明城市资格城市，龙港书芳苑等3个社区被评为全国和谐示范社区。积极开展党的群众路线教育实践活动，围绕为民务实清廉主题，主动“照镜子、正衣冠、洗洗澡、治治病”，立行立改解决“四风”突出问题。会议减少11%，文件减少21%，部门一般性支出下降40.8%，“三公”经费下降34.4%。我市转职能、转方式、转作风的做法和严肃查处群众身边的腐败问题等经验受到中纪委监察部充分肯定。

　　每季度向市委常委会报告工作。自觉接受市人大及其常委会的法律监督、工作监督和市政协的民主监督。坚持定期向市人大报告工作、向市政协通报工作，认真听取采纳人大代表、政协委员意见建议，建立了经常性工作协商协调机制。办理人大代表建议78件、政协提案515件，办复率、见面沟通率100%，满意和基本满意率99.5%，落实和基本落实率69.3%。推进“六五”普法和法治城市建设，便民服务网络覆盖乡镇(街道)，社区矫正管理全程信息化。社会治安防控体系不断加强，刑事案件下降13%，荣获平安辽宁建设先进市。应急管理体系逐步完善，突发事件处置能力不断增强。矛盾纠纷调处力度加大，信访积案化解率73%，荣获全省综治工作先进市八连冠。加强审计监督，完善行政制约机制。严厉打击食品药品违法行为，确保群众饮食用药安全。坚持党政同责、一岗双责、齐抓共管，推动安全生产“五个全覆盖”和“打非治违”常态化，荣膺全省安全生产先进市六连冠和全国安全生产月活动先进市七连冠。

　　各位代表：过去的一年，我市经济经受住了“三期叠加”和严重旱灾的重大考验，顶住了持续下行的巨大压力，保持了健康发展的基本态势，奠定了“富民升位”的坚实基础。这是省委、省政府和市委正确领导的结果，是市人大和市政协监督支持的结果，是全市人民共同奋斗的结果。成绩属于全市人民!在此，我代表市政府，向全市各族人民，向人大代表、政协委员和老领导、老同志，向各民主党派、工商联、无党派人士、人民团体和社会各界人士，向驻葫部队指战员、武警官兵、公安民警和中省直单位，向所有关心支持葫芦岛建设发展的朋友们，表示崇高的敬意和衷心的感谢!

　　回顾过去一年工作，我们清醒地看到，我市发展仍然面临许多困难和挑战。主要表现在：一是宏观经济环境仍然严峻，企业效益不佳，政府进入还债高峰期，偿债压力较大。二是产业结构调整比较艰难，项目建设相对滞后，有效投资明显不足，园区的产业聚集效应尚未形成。三是民营经济活力仍显不足，融资渠道狭窄，规模普遍较小，产业层次较低。四是城镇化水平不高，就业和民生改善压力较大。五是群众关注的空气污染、环境保护、食品安全等问题压力增大。六是少数干部仍然存在慢作为、不作为、缺少担当精神等问题，发展软环境仍需进一步改善。

　　对于这些问题，我们将高度重视，认真解决。

　　二、2015年主要工作安排

　　2015年，是“十二五”收官之年，是全面深化改革的关键之年，也是全面推进依法治国的开局之年。从当前形势看，宏观经济正在进入新常态的深度显现期，我市经济中低速增长的态势短期难以改变。但也面临着世界经济逐步复苏、政策效应逐步显现、改革红利逐步释放等重大机遇。由此判断，我市仍处于大有可为的战略机遇期、结构调整的加速期、新型产业的成长期。我们要把握经济新常态的阶段性特征，把改革红利转化为转型动力，把下行压力转化为增长动力，努力开创改革发展的新局面。

　　2015年，政府工作的总体思路是：全面贯彻党的十八大、十八届三中、四中全会和习近平总书记系列重要讲话精神，认真落实中央和省委、市委经济工作会议的决策部署，坚持依法治市、稳中求进工作总基调，坚持以提高经济发展质量和效益为中心，坚持以转方式调结构为重点，主动适应经济发展新常态，保持经济运行在合理区间，狠抓改革攻坚，突出创新驱动，强化风险防控，注重环境保护，加强民生保障，促进经济平稳健康发展、社会和谐稳定。

　　2015年的主要预期目标是：地区生产总值增长6%，公共财政预算收入增长2%，固定资产投资增长6%，社会消费品零售总额增长11%，城镇居民人均可支配收入增长8%，农村居民人均可支配收入增长9%。同时，及时进行底线监测，适时进行区间调控，确保经济运行处于合理区间。

　　为实现上述目标，重点抓好九个方面工作：

　　1、抢抓新一轮东北振兴机遇，继续深化改革、扩大开放

　　推动改革攻坚。加快简政放权，全面实行“三证合一”制度，减少行政审批事项，简化办事流程，提高办事效率。深化政府机构改革，规范机构设置，优化行政效能。推进事业单位分类改革，完成承担行政职能和从事生产经营活动的事业单位改革任务。推进财税体制改革，加强预算管理，健全预算体系，完善政府债务管理制度和风险预警处理机制。推进金融体制改革，扩大信贷融资和非信贷融资，引导企业开展直接融资，培育多层次资本市场，支持实体经济发展。统筹推进国资国企、医药卫生、户籍等领域改革。

　　突出创新驱动。建立产学研协同创新机制，加快“龙湾硅谷”和“海岸中关村”2个科技成果转化基地建设。完善高新技术企业扶持措施，孵化科技型中小企业，推动高新技术企业倍增发展。加快结构调整，推进城区老工业区搬迁改造和独立矿区整体搬迁改造，深化与央企、民企、沪企“辽宁行”合作项目对接，推进3000亿元产值新型石化产业基地建设，促进传统优势产业向价值链高端延伸。

　　深化沿海开放。加快招商选资，以京津冀、长三角、珠三角为重点，以园中园为依托，强化区域性联合招商、产业链招商，促进招商项目集团化落户。加快推进港区融合、产城融合，启动绥中港区对外开放。借鉴保税区、自贸(港)区运营模式，整合园区产业定位，深化复合型园区建设，推动园区资源共享、要素互通、优势互补，形成一体化发展格局。

　　2、推进工业结构调整，提升工业经济运行质量

　　全力推动重大项目建设。强化包保责任制，加大企业扶持力度，促进有效投资增长。规划投资亿元以上项目216个，总投资2910亿元，当年完成投资482亿元。北京国浩磁流变液、大唐热电等80个项目开工建设，完成投资198亿元;徐大堡核电等85个项目加快建设，完成投资223亿元;中泰6万吨焊材、高桥风电等51个项目建成投产，完成投资61亿元。深入实施工业五项工程。抓好创新示范企业50家，规模以上企业增长10%。推进兴城粉末冶金、辽宁合众凯达等企业升级，完成葫芦岛有色重组。深化宏跃集团与江西铜业合作，建设东北最大精铜冶炼基地。推进海外并购项目8个，促进企业低成本提质扩张。推进产业集群梯次发展，现有6个产业集群企业突破1300家，提高产业集群丰厚度。启动膨润土、紫砂2个产业集群建设，引进企业8家，力争两年内形成亿元产值规模。

　　加快民营经济发展。实施大众创业、万众创新工程，充实市县两级创业基金，完善街道和乡镇普惠制创业辅导，激发市场活力，推动混合所有制经济和民营经济发展。健全融资担保机制，推动中小微企业加快发展。拓宽融资渠道，推动企业上市，培育更多企业到新三板、辽股交登记挂牌。发挥市中小企业服务中心作用，加强对科技型、成长型工业企业的定向扶持、专项服务，降低经营成本，以创新推动创业，力争新增登记企业2.4万家。

　　3、转变农业发展方式，壮大县域经济实力

　　推进农业结构调整。实施“富民升位”农村小康工程，启动旱作农业、水果、干果、海洋牧场、设施农业、畜禽养殖“6项增收工程”，发展高效、绿色、节水农业，提高抵御风险和增收能力。用三年时间，打造沿海设施农业带、现代渔业带和水果产业带，打造内陆旱作农业区、干果经济区和畜禽养殖区，形成“三带三区”农业产业新格局。

　　推进农业现代化。以农村土地适度规模经营为方向，扩大土地确权规模，规范土地流转行为，提高土地流转率。培育新型农业经营主体，新增带动100户以上的农民合作社30个，新增经营100亩以上的家庭农场30个。实施龙头企业、农业品牌和科技推广工程，新增省级重点龙头企业5家、市级龙头企业10家，10个农产品深加工项目全部投产，力争3个县(市)全部成为省一县一业示范县。

　　推进宜居乡村建设。以“我们的乡村·我们的家园”为主题，以农村产业发展为引领，以“环境整洁，设施完善，生态优良，传承历史，富庶文明”为标准，突出优化农村生态环境、提升农民生活质量、发展农村生态经济、打造乡村特色文化4个重点，完成32个示范村和251个达标村建设任务，着力建设宜业宜居宜游的美丽家园。

　　4、推动服务业加快发展，提高城区经济效益

　　加快服务业集聚区建设。加快金岸龙湾旗帜型服务业集聚区建设，以“十里银湾”集聚带为核心，推动龙港和连山商贸集聚区融合发展。推进兴城滨海旅游、大红门物流、打渔山科技服务等3个集聚区建设，前所风情小镇、觉华岛旅游度假区等15个亿元以上项目开工建设，兴隆大家庭、奥特莱斯等13个项目主体完工，五金机电城、银象宁远城、瀚沃商贸城等12个项目竣工营业，限额以上服务业企业增长10%。

　　加快发展电子商务。以跨境电子商务平台为依托，打造虚拟产业集群，建设中国北方“电子口岸”，推动服务业转型升级。两年内，全市电商企业突破1万家，交易额突破100亿元，实现倍增发展。吸引品牌快递企业落户，提高特色产品网销量，促进要素跨境流通，拉动物流产业快速发展，推动贸易便利化。

　　加快培育新兴服务业态。发展信息技术、金融、政府服务等领域的服务外包，推动服务业向价值链高端延伸。引导餐饮业特色化、大众化、健康化经营，提高商场夜间销售额。推进民生银行开业运营，葫芦岛银行新设外埠分行。引导刚性需求，盘活存量资源，发展商业、工业、旅游和养老地产，促进房地产业健康成长。

　　5、加大资源整合力度，大力发展文化旅游产业

　　推进全市旅游一体化。启动龙湾海滨至兴城海滨国家5A级景区和沿海国家湿地公园创建工作，整合全市旅游资源，实施全域化旅游战略。成立旅游发展集团，市场化运作，企业化经营，统筹景区管理和演艺业务。探索葫芦岛旅游一卡通，实现全地区景区景点互联互通。依托辽冀蒙八城市旅游联盟，推进域内外旅游合作机制，打造大区域旅游合作体系。

　　开发新型旅游产品。加快徐大堡、永安堡、凉水井子等10个旅游名镇名村建设，打造风情小镇、特色村落。发展海洋、森林和温泉旅游，推进觉华岛、龙湾海滨、兴城海滨、东戴河“四点一线滨海观光游”，推出首山、灵山、虹螺山、白狼山“4山森林氧吧游”，打造丽汤温泉、汤上温泉小镇、富都温泉乐园、百大温泉乐园“4个温泉游”。发展康复和养老旅游，建设龙湾、邴家湾、止锚湾3个康复旅游中心，开发附加值较高的旅游纪念品，形成新型文化旅游品牌。

　　发展文化创意经济。继续办好泳博会、泵博会和菩提文化旅游博览会，加强邴家湾创意产业园和觉华岛菩提园建设，打造会展经济产业集群。制定创意产业政策，培育创意生态，打造创意品牌，建设“新型智库”，推进创意生态城市发展。设立文化创意政府奖葫芦岛文艺奖，培育跨演艺、音乐、文学、影视、时尚行业的创意团队，繁荣文艺创作和演艺产业，逐步建立文化创意产业链。

　　6、加快美丽葫芦岛建设，推进新型城镇化进程

　　优化城市功能。积极推进政府与社会合作投资模式，化解存量债务和基础设施及市政公用事业建设的融资难题。完善城乡路网，大修兴凌线，开工建设绥中港疏港高速公路，开通兴建高速客运线路。提升供热供电质量，茨齐路、东城区并网集中供热，提高城区供热质量;新建4座变电站，推进农网改造升级。加快猴山水库建设，从根本上解决东戴河新区用水瓶颈问题。深化“我们的城市·我们的家园”城区环境整治，新建滨海公园，龙湾海滨至兴城海滨木栈道全线贯通，实现绿化、美化、净化、亮化水平整体提升。

　　建设生态城市。实施青山工程，治理闭坑矿和破损山体，开展非法侵占林地清理排查，严厉打击破坏森林资源违法行为。加强植树造林，扩大森林覆盖率，确保晋级国家森林城市。实施碧水工程，全面治理五里河、茨山河等5条河流水质，完成连山、建昌和方大化工污水处理厂建设，严查沿海排污，整治非法排污，实现水污染物减排目标。实施蓝天工程，升级改造燃煤锅炉，公交车油改气，专项整治工业大气污染、扬尘污染、尾气污染和雾霾天气，全面完成“十二五”总量减排任务。

　　推进新型城镇化。完善葫兴一体化规划，加快基础设施建设，推动产业互联互通，实现主城区与兴城城区的链接。扩充明星城镇规模，推动中心城区、县城、新区新市镇和重点村镇协调发展，打造沿海城镇带。加快乡镇规划修编和户籍制度改革，依托京沈高速、102国道和兴建高速，发展通道经济，引导人口向县城和城镇集聚，推动山水乡恋与城市文明融为一体的新型城镇化。

　　7、发展社会事业和实施重大民生工程，增强人民群众幸福感

　　加强人才、就业和社会保障工作。创新人才引进、培养、选拔和录用机制，新增实名制就业4万人，城镇登记失业率控制在3.8%以内。加强劳动技能培训，转移就业农村劳动力30万人，加快社保扩面，实现养老、医疗、失业、工伤和生育保险参保人数稳中有升，城乡最低生活保障标准、农村五保对象救助标准分别提高10%左右。

　　发展各项社会事业。加快教育现代化步伐，打造20所城乡普惠性幼儿园，建设96所标准化学校，完成中职学校资源整合，促进普通高中教育特色发展。狠抓师德师风建设，整治有偿补课和接受学生财物问题。发展群众文化事业，培育民营文化团体，推进文化场馆向公众开放。活跃体育赛事，发展赛事经济。开展全民健身活动，提高全民健康水平。

　　今年，我市将进一步加大民生投入，集中办好与群众生活密切相关的十大民生工程。一是供水安全工程。实现青山水库向城区居民供水，确保城市居民饮水安全。二是道路畅通工程。启动城市外环路建设，完成动迁任务，修建天化路，打通城区道路瓶颈。三是管网改造工程。全面启动老城区地下管网改造，保障老城区上下水通畅。四是养老设施工程。建设60家农村幸福院，改造6家农村中心敬老院，500张床位老年公寓主体完工。加强养老服务网络建设，为1万名老年人购买一键呼叫的专用手机。五是公园提升工程。实施龙湾公园改造，完善景观结构，改良滨水空间，增设公共设施，为市民提供良好休憩场所。六是住房保障工程。改造城市棚户区3518户，改造农村危房1500户，新建保障性住房400户，发放廉租房租赁补贴200户，援建农村贫困母亲安居住房100户。七是工资津贴提升和社保提标扩面工程。提高机关事业单位基本工资标准和企业退休人员基本养老金，建立正常调整和增长机制。将全市小学和初中班主任津贴提高到每月100元。提高城乡居民养老保险、医疗保险等财政补贴标准，扩大对农村妇女“两癌”筛查的补助范围。八是文化场所建设工程。比基尼广场全面竣工使用，增加广场演艺功能。完成93家社区书屋和广播电视“户户通”工程建设。九是公共卫生惠民工程。推进兴城、建昌县级公立医院改革，开工建设市妇幼保健和计划生育服务中心。建立重特大疾病医疗救助制度。低保对象、特困供养人员基本医疗住院救助比例提高到70%。普及城乡居民健康卡，全面实施新农合和大病保险“一站式”报销服务。十是扶贫帮困工程。创新扶贫开发机制，确保7万农村贫困人口脱贫。实施贫困残疾人助行和家庭无障碍改造900户，对经济困难的失能老人给予每人每月100元生活补贴。

　　8、加强民主法制和精神文明建设，推进依法治市进程

　　推进民主法制建设。贯彻党的十八届四中全会精神，忠实履行宪法和法律赋予的职责，运用法治思维谋划发展，运用法治方式规范管理，运用法治手段化解矛盾，全面推进依法治市进程。定期向市委常委会报告工作。自觉接受市人大及其常委会的法律监督、工作监督，定期向人大报告工作，全面执行人大及其常委会的决议和决定。积极支持市政协履行政治协商、民主监督和参政议政职能。听取各民主党派、工商联和无党派人士意见建议。认真负责办理好人大代表建议和政协提案。加强审计监督，接受新闻舆论和社会公众监督。深化“六五”普法，培育法治信仰，倡导法治精神，建设法治文化。

　　加强和创新社会管理。推进平安葫芦岛建设，加强社会治安立体防控体系建设，完善“天眼工程”，新增治安监控摄像头5000个。增加安全防范措施投入，规范社区和繁华街区武装巡逻，增强人民群众安全感。加强互联网管理，维护网络和信息安全。创新食品药品监管模式，实行最严格的安全监管制度，保障“舌尖上的安全”。

　　加强安全生产隐患排查治理，遏制重特大安全生产事故。完善防灾减灾应急保障体系，提高预防预警和应急处置能力。加强民族宗教工作。发挥人民调解、行政调解、司法调解作用，营造和谐稳定社会环境。

　　深入推进精神文明建设。弘扬社会主义核心价值观，深化全国文明城市创建活动，开展社会公德、职业道德、家庭美德、个人品德教育，提高市民文明素质和社会文明程度。开展“葫芦岛好人”、“诚信示范单位”等活动，用身边典型引领社会风尚，塑造现代城市精神。深化双拥共建，加强国防教育和国防后备力量建设，推动军民融合深度发展，争创省双拥模范城六连冠、全国双拥模范城五连冠。

　　9、坚持为民务实清廉，加强政府自身建设

　　建设法治政府。推行法无禁止皆可为的“负面清单”、法无授权不可为的“权力清单”和法定职责必须为的“责任清单”，全面依法履行职能。敬法畏纪，遵规守矩，权责统一，严肃约谈问责，严查违法行政。深化政务公开，拓宽沟通渠道，保障公民知情权、参与权、表达权和监督权的落实，让权力在阳光下运行。

　　建设创新政府。坚持科学决策、民主决策，规范办事程序，优化行政流程，提高行政效能。转变工作作风，严格目标责任制，整治不作为行为，弘扬担当精神，激发干部干事激情，增强公共服务能力。

　　建设清廉政府。强化廉政监察、执法监察、效能监察，巩固和拓展教育实践活动成果。深入反“四风”，持续抓整改，厉行勤俭节约，严控“三公经费”，强化预算管理，做到干部清正、政府清廉、政治清明，营造风清气正的政治生态。

　　建设为民政府。树立群众观念，深入实际，深入群众，兢兢业业，踏踏实实，解难题，办实事，把更多精力用到服务群众上，把更多资金投入到改善民生上，努力提高人民群众的幸福感。

　　各位代表!新常态蕴涵新机遇，新挑战需要新作为。让我们在市委的坚强领导下，以新状态适应新常态，以新作为推动新发展，振奋精神，勇往直前，齐心协力，埋头苦干，为建设生态宜居的沿海经济强市而努力奋斗!
